# Accidental Poisoning with Arsenic Successfully Treated with the Hydrated Peroxide of Iron

**Published:** 1845-02

**Authors:** J. J. Kelso

**Affiliations:** Lisburn


*Accidental Poisoning with Arsenic successfully treated with the Hydrated Peroxide of Iron. By J. J. Kelso, M.D., Lisburn.* Though a wide-spread skepticism regarding the efficacy of the hydrated peroxide or sesquioxide of iron, as an antidote to poisoning with arsenic, most undoubtedly, and, considering the number of different substances that have successively been proposed for this purpose, only to be rejected each in turn as utterly valueless, not unnaturally, obtains; still, from repeated experiments, both of a physiological and chemical nature, and more especially from actual experience of the preparation in cases of poisoning in the human subject, its power of converting the white oxide of this metal into an insoluble compound—the arsenite of iron—and thereby rendering it innoxious in its effects, as well in man as in the lower animals, seems at present to be pretty well established, and very generally admitted by many members of the profession—and, among others, by Christison (*Dispensatory*, p. 184) and Pereira (*Elements of Materia Medica*,) on whose authority we are accustomed, not without sufficient reason, to repose. It may not, perhaps, be too much to affirm, that on the whole a growing faith in favor of the counteracting influence of the rust of iron, either in the state of magma or dry powder, in instances where the arsenic has been ingested in poisonous doses, at least under certain conditions, and to a certain extent, is evidently and very reasonably beginning to manifest itself. Be this as it may, the following case presents, I am strongly inclined to think, a very striking exemplification of the good effects of the remedy.

Mary C——, a fine, healthy, intelligent little girl, of about eight years of age, swallowed, on the 18th of May last, a quantity of arsenic, or at least what then was believed to be such, which had been designedly mixed with some oatmeal, and set aside in a particular place for the purpose of poisoning rats.

The facts of this very interesting case are briefly as follows: C——, the child's father, it appeared, purchased on the preceding evening, at an apothecary's shop in Lisburn, a pennyworth of white arsenic, in powder, which I have every reason to believe might have amounted in quantity to upwards of an ounce at least. This, indeed, is admitted by the gentleman himself who vended the article, and whose recollection of the transaction, when questioned on the subject, enabled him besides to verify the still more important fact of the substance itself being arsenic—the white oxide, of course, of the shops. On reaching home, he took of the substance thus procured, as near as he could estimate, rather more than the one-half, and having mixed it with more than double its bulk of dry oatmeal, he distributed it equally on two pieces of broken crockery, which he placed, at bedtime, in separate places in an apartment of the house, for the purpose of destroying the rats, with which it is, it appears, infested. The operation, he says, was performed in the presence of his wife and the girl in question, in common with two or three others of the more advanced members of the family. He further particularly states his having, at the time, distinctly and repeatedly mentioned in the presence—and hearing, of course—of the family group, who were closely congregated in witnessing the interesting procedure around him, the fact of the powder which he was commixing being very poisonous in its charactcr, and that its object was for the destruction of rats. However that may be, his wife in the morning, took up, for the sake of greater security, both pieces of crockery containing the mixture indicated, or rather, the portions that remained unconsumed by the vermin, which she says was considerable. One of these she very properly placed in a spot beyond the possibility of reach by any of the children, while the other, quite unaccountably, she left in a place that was easily accessible by almost all. The consequence was, that, not long after, our little patient, in her rambles, came upon the dish containing the residue of the poisonous mixture, and, in forgetfulness or ignorance of what it really was, at once consumed the entire contents.

This occurred at about 11 o'clock, A. M., and in rather less than an hour and a half after, I saw her. Her mother, who seemed in great distress, informed me that the first intimation she had of the startling fact of the child having eaten the poisonous compound, was in commencing to vomit on being presented with some food. She immediately had recourse, she further stated, to the popular remedy of melted butter, which she administered in considerable quantity. This had the effect of exciting rather free vomiting, which had been repeated, it appears, two or three times. Her symptoms then were these: Marked listlessness and indisposition to reply to questions; pulse about 120, weak, and rather unequal, with occasional indications of fluttering; respiration not irregular, but rather slow, and imperfectly performed; and a feeling of sickness or nausea, with an inclination every now and then to vomit. When asked if she had any feeling of heat or pain in the throat or stomach, she gave me distinctly to understand that she experienced, in some degree, both of these sensations. The pain in the epigastric region was evidently increased on the application of the hand, and she then became fearful, and complained of the procedure. The pupil of either eye, but especially the right one, was rather remarkably, and, in cases of this kind, I believe, unusually influenced. Alternate dilatations and contractions, in rather rapid and extreme degree, were observed to occur; these were evidently still further increased on the presentation of a lighted candle, or the tip of the forefinger motioning to and fro. There was no other derangement of function deserving mention, vital, physical, or mental.

On questioning the little patient herself, she freely confessed, as she had previously done, to the fact of having eaten the powder containing the deleterious ingredient—not, she added, as plainly as she could speak it, by licking it up with her tongue, but by introducing it into her mouth, pinch by pinch, between her fingers and thumb. On further interrogation, she equally admitted having been told, on the preceding evening, that the substance then being mixed with the meal was "poison," and what its particular object was. Her recollection of all this seemed to be quite distinct, but on still further inquiry I satisfied myself that she had not been cautioned as to its deleterious properties in reference to herself. The impression she had evidently imbibed was, that what is poisonous to the lower animals might be perfectly innocuous in reference to the human species. Want of proper instruction, or observation, or experience, rendered her incompetent to the formation of the general notion, that what is deleterious to some of the higher species of brute life is usually equally so, under certain conditions, to man. Locke, doubtless, would have regarded this incident as a happy exemplification of his well-known doctrine concerning the origin of ideas; at all events, the source of error thus became at once apparent.

The conclusion to which all these facts tend is inevitable, that the case was one of poisoning by arsenious acid. In order, however, to obviate any objection that might occur, respecting the perfectly satisfactory character of the evidence by which it is sustained, it may not be improper here to mention, that I satisfied myself, from an inspection of the substance which remained in the packet already referred to, of the fact of its really being what the gentleman who vended it stated it to have been, the common or white oxide of arsenic of the shops.

However, the only substances in the shape of antidotes that were accessible where the accident occurred, being chalk and magnesia, I prescribed a small teaspoonful of each in sweet milk every fifteen minutes.

At about half-past 4 o'clock I again visited the patient. Her condition then was this: there were evidently much sickness at stomach and indisposition to action; she had vomited twice or thrice rather copiously, and the exhaustion occasioned in consequence was clearly indicated in the general appearance presented by the countenance. The eyelids drooped languidly, concealing to some extent the eyeballs; and the features generally were relaxed, so as to give the countenance a rather expressionless appearance; the involuntary movements of the iris of either eye had in a great measure disappeared, indicating the re-establishment of the impaired nervous energy of the brain, to which they were evidently referable. The pulse was 130, weak and indistinct; epigastric region considerably distended, and acutely painful on pressure; and the patient complained of a burning sensation in the course of the œsophagus and stomach. There was some thirst, but no evacuation either of the bowels or bladder. Altogether, the phenomena at this time appeared most unpropitious; she was ordered a small teaspoonful of the hydrated peroxide of iron in water every forty minutes; gum water *ad libitum.*

The result of the case may be told in a very few words. In about three hours after the first administration of the remedy, which, with the exception of the second or third dose, was well retained by the stomach, the more prominent and violent phenomena began sensibly to yield. In twelve hours more she was considered to have braved all the peril, and to be in a state of convalescence. She consumed in all, perhaps, about from three to four ounces of the ferruginous preparation, a proportion greatly in excess to the arsenic we may suppose to have been retained by the stomach after the frequent and rather copious vomitings that occurred. Considering the dose of arsenic ingested as between one and two drachms—and I think it could not have been less—there would probably be rejected in the fluids vomited fully three-fifths, leaving in the stomach little short of a scruple, perhaps, to be acted on by the iron. Hence the remedy compared with the poison must have been considerably in excess. I shall only further add, that the diarrhœa which supervened passed off in little more than forty-eight hours, requiring only some castor oil and demulcent drinks.

The length to which the details of this case have run, prevents me from adverting, in this place, to the results of recorded experience in favor of the rust of iron, either in the moist or dry state, as an antidote to poisoning by arsenic. On consulting the periodical medical literature of these countries for the last eight or nine years, several instances strikingly illustrative of its efficacy are to be found. Within this period, eight or ten such instances may be found reported in the pages of "the Medical Gazette," (vol. xv., p. 448; xvi., p. 832; xix., p. 177;) "Medico-Chirurgical Review," (vol. xxxiii., p. 534;) and "the Lancet," (vol. xxxviii., p. 625; xl. p. 275.) For the details of these, some of them very interesting cases, I must content myself by referring to those publications.

Before concluding, it may not be improper to mention the method employed by me in obtaining the preparation of iron in question. This consisted simply in adding to the liquor of chloride of iron, water of ammonia, collecting the precipitate, dissolving in water, and finally throwing it on a filter. The object of wasting the peroxide of iron precipitated in this manner is to remove any excess of ammonia with which it may be charged. This I consider worthy of very particular observation, as the irritating effects of the alkali in any quantity must obviously, where the local irritation or inflammation is considerable, prove highly prejudicial, and be a source of further mischief.—*Bulletin of Med. Science*, from *Lon. Lancet.*

